# Low dose ribosomal DNA P-loop mutation affects development and enforces autophagy in Arabidopsis

**DOI:** 10.1080/15476286.2023.2298532

**Published:** 2023-12-29

**Authors:** Thiruvenkadam Shanmugam, Palak Chaturvedi, Deniz Streit, Arindam Ghatak, Thorsten Bergelt, Stefan Simm, Wolfram Weckwerth, Enrico Schleiff

**Affiliations:** a Molecular Cell Biology of Plants, Institute for Molecular Biosciences & Buchmann Institute for Molecular Life Sciences, Goethe University Frankfurt, Frankfurt am Main, Germany; b Molecular Systems Biology (MOSYS), Department of Functional and Evolutionary Ecology, University of Vienna, Vienna, Austria; c Institute of Bioinformatics, University Medicine Greifswald, Greifswald, Germany; d Vienna Metabolomics Center (VIME), University of Vienna, Vienna, Austria; e Frankfurt Institute for Advanced Studies, Frankfurt am Main, Germany

**Keywords:** rRNA mutation, plant development, CRISPR, dosage compensation, autophagy

## Abstract

Arabidopsis contains hundreds of ribosomal DNA copies organized within the nucleolar organizing regions (NORs) in chromosomes 2 and 4. There are four major types of variants of rDNA, VAR1–4, based on the polymorphisms of 3’ external transcribed sequences. The variants are known to be differentially expressed during plant development. We created a mutant by the CRISPR-Cas9-mediated excision of ~ 25 nt from predominantly NOR4 ribosomal DNA copies, obtaining mosaic mutational events on ~ 5% of all rDNA copies. The excised region consists of P-loop and Helix-82 segments of 25S rRNA. The mutation led to allelic, dosage-dependent defects marked by lateral root inhibition, reduced size, and pointy leaves, all previously observed for defective ribosomal function. The mutation in NOR4 led to dosage compensation from the NOR2 copies by elevated expression of VAR1 in mutants and further associated single-nucleotide variants, thus, resulting in altered rRNA sub-population. Furthermore, the mutants exhibited rRNA maturation defects specifically in the minor pathway typified by 32S pre-rRNA accumulation. Density-gradient fractionation and subsequent RT-PCR of rRNA analyses revealed that mutated copies were not incorporated into the translating ribosomes. The mutants in addition displayed an elevated autophagic flux as shown by the autophagic marker GFP-ATG8e, likely related to ribophagy.

## Introduction

**Figure 1.**
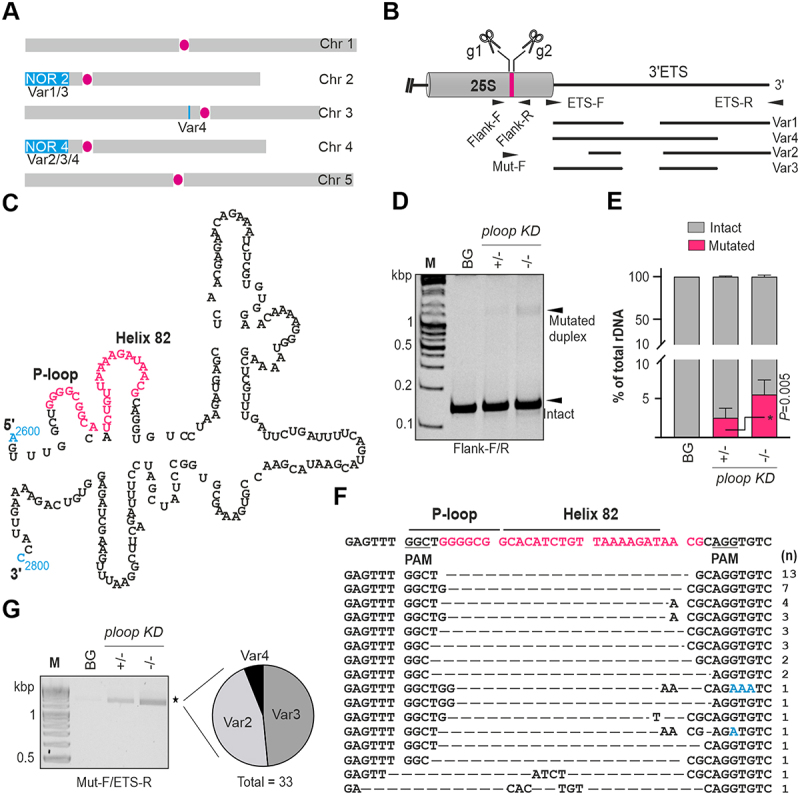
Targeted mutagenesis of 25S rDNA led to mosaic mutation patterns on chromosome 4. (A) Schematical distribution of the rDNA copies on five Arabidopsis chromosomes and their associated VAR polymorphic rRNAs. (B) 3’-ETS polymorphism in the *A. thaliana* col-0 accession and the oligonucleotide’s binding positions for the detection and identification of mutated VARs in the genome. (C) Location of the targeted bases, consisting of the P-loop and helix-82 regions, in the secondary structure of the 25S LSU ribosomal RNA [9] is indicated in magenta. Numbers 2600 and 2800 in cyan denotes the positions of nucleotides in 25S rRNA (D) Heteroduplex PCR products of three genotypes electrophoresed under 15% native PAGE conditions. (E) Quantification of the mutated and intact rDNA copies of the PCR products analysed in panel C (*n* = 6 linearly increasing PCR cycles). Data are shown as means ± SD among the total % of rDNA. The P-value indicates the statistical significance with the unpaired student t-test. (F) Cloning and sequencing of the heteroduplex PCR products of panel C resulting in mosaic mutation patterns in the rDNA. (G) Electrophoresis of the PCR products on 2.5% TTE gels from the three genotypes; PCR was carried out using the mut-F and ETS-R oligos, followed by cloning and sequencing of 33 independent clones – their linked VAR subtypes are summarized.

Ribosomes are complex machineries required for protein synthesis in all life forms [1]. In eukaryotes, the rate of their production depends on the developmental stages and requires up to 60% of the cellular ATP [2]. The major structural components of the ribosomes are the ribosomal RNAs [3,4] and ribosomal proteins (RPs) [5]. In *A. thaliana*, the mature ribosomes are composed of four ribosomal RNAs (18S, 5.8S, 25S and 5S) and all of them other than 5S are encoded in the nucleolar organizing regions 2 and 4 (NOR2 and NOR4) of the telomeric regions of chromosome 2 and 4, respectively. The selective NOR silencing after early seedling development stages is achieved through position-dependent effect specific to NOR2, mediated by local chromatin modificiations [6]. These 45S rDNA copies are arranged in a tandem fashion with intermittent intergenic spacers. Based on the length of the polymorphisms at the 3’-ETS regions of these rDNA copies, they are classified into four types: VAR1, 2, 3 and 4. Here, NOR2 contains only VAR1 and VAR3, while NOR4 contains only VAR2, 3 and 4 [7,8]. A few copies of the VAR4 type rDNA copies are also associated within the 5S repeats of chromosome 3, close to the centromeric region (Figure 1A) [10].

The 32 ribosomal small subunit (RPS) and 48 ribosomal large subunit (RPL) proteins are associated with the rRNA in mature ribosomes [11]. In addition, eukaryotic ribosomes transiently associate with ribosome biogenesis factors (RBFs) that aid ribosome maturation (termed ‘ribosome biogenesis’) in the nucleolus, nucleoplasm, and cytoplasm [12]. In plants, more than 200 RBF factors are discussed to be involved [13,14].

Throughout embryonic and reproductive plant development, the variable metabolic demands are correlated with differing ribosome requirements. Thus, the plethora of RNA and protein components facilitates wide-ranging ribosomal heterogeneity [15] as documented for the 80S ribosomes in Arabidopsis [16]. Therefore, many of the RPs and RBFs are encoded by multiple paralogues that are under differential promoter expression control in terms of growth stages, tissue specificity and hormonal control [17]. Moreover, the genetic diversity of the RP gene families in the plant kingdom is considerably higher than other model eukaryotes, by as much as ten-fold, in the wake of genome duplication events [18]. In Arabidopsis, 102 and 146 genes code for the RPS and RPL proteins, respectively [19,20]. The compositional variations of the ribosomes by the differential expression of RPs [21] and the sequence polymorphisms of the ribosomal RNAs [7,22] are further factors facilitating heterogeneity. In addition, the rRNA-based regulation of translation is regulated by the presence or absence of 2’-O-methylation (Nm) of the ribose moiety and the pseudouridylation (Ψ) of uridines of the rRNA [23,24].

In Arabidopsis, mutants impaired in ribosome biogenesis show two common phenotypic classes. The majority of mutants of known RPs and of essential RBFs are lethal for the female gametophyte or embryonic development [13,25]. These cells depend on high ribosomal activity and, significant delays in the maturation or assembly of the ribosomes can lead to cell division defects resulting in cell death. The mutation of non-essential RPs and RBFs can exhibit a variable scope of non-lethal disorders in terms of root growth and leaf patterning defects [26]. These phenotypes, although affecting the overall fitness of plants, still permit reproduction.

In addition to the variance in composition, a biased expression of the population of rRNAs with skewed single-nucleotide polymorphisms has been reported that may also lead to altered ribosome translation potential in other organisms [27–32]. In Arabidopsis, the chief source of single-nucleotide variants (SNV) in the rRNA subpopulation is their biased distribution in the genomic locations of NORs and, thereby, their associated rRNA variants that are known to be variably expressed. The unique expression profile of specific VARs at different stages of growth and development is associated with the chromatin-mediated silencing of either NOR2 or NOR4. VAR1 is expressed during the early stages of seed formation and seedling growth stages. The subsequent silencing of NOR2 leads to the lack of VAR1 rRNA type during the adult growth stages [7,8,22]. However, the consequences of the variability and the mutation of specific variants of ribosomal RNAs on the phenotypic fitness of plants remain elusive.

We have explored the phenotypic fitness of mutants generated by the CRISPR-Cas9-mediated excision of a regulatory motif at the 3’ side of the 25S rDNA. Despite their target being multicopy rDNAs, the guide RNAs induced mutations only in a fraction of their rDNA target copies, with the mutation leading to typical ribosomal mutation-like growth defects. We show the fitness costs of mutating critical rDNA copies on plant growth and development. Our results have implications for understanding the selective influence of ribosomal DNA copies on plant growth and development, as well as for the definition of quality control mechanism in plants.

## Results and discussion

### CRISPR-Cas9 mutagenesis of the LSU ribosomal RNA element, P-loop, resulted in the selective targeting of the chromosome 4 copies

CRISPR-Cas9 targeting of the 25S rDNA was employed to excise ~ 25 bp in multiple rDNA copies to deduce the effect of rDNA mutation on the phenotypic fitness of Arabidopsis. The two guide RNAs were poised to induce mutations close to the polymorphic 3’-ETS region (Figure 1B). Structurally, the excised region disrupts two critical elements, the P-loop and helix 82 (Figure 1C) which are positioned near the E/P/A site of the ribosomes and in the vicinity of the N-terminus of RPL29 alongside RPL10 [33]. The transformation of the CRISPR-Cas9 expression cassettes resulted in an altered leaf morphology containing pointy rosette leaves (T1 plants: 7 of 14 plants (7/14); Figure S1A). In subsequent generations, the T2–4 progenies carried mutations based on the specific appearance of heteroduplex PCR bands with their accompanying phenotype (Figure S1B, C). A single progeny lacking the Cas9 expression cassette was selected to avoid incessant mutations (T2-4-5 in Figure S1D). The heterozygous mutations are segregated with a 1:2:1 ratio into WT-like (BG), heterozygous (*ploop KD* ±) and homozygous (*ploop KD* -/-) for the mutated *ploop* allele copies. The proportion of rDNA copies carrying mutations was determined by heteroduplex PCR. The ratio between the intensity of the mutated PCR band relative to the intact rDNA band was estimated through targeted loci-flanking PCR (Figure 1D) resulting in specific mutated duplex bands occurring for the *ploop KD* plants. The analysis showed that 2.5% and 5.5% of the total rDNA copies were perturbed in the heterozygous and homozygous conditions, respectively (Figure 1E). The patterns of the mutations were mapped by the cloning and sequencing of the heteroduplex bands (*n* = 44). The Cas9 excisions were predominantly deletions between the two guide recognition sites. The complete excision of the target region with a one-bp excess on guide 2 was dominant (13/44), followed by a one-bp excess on guide 1 and a two-bp excess of guide 2 (7/44) and a three-bp excess on guide 2 (4/44). The frequency of all other cases was below 10% of the total. Overall, the CRISPR-Cas9 targeting of the 25S rDNA using dual-guide RNAs resulted in the stable inheritance of mosaic mutation patterns in the ribosomal DNA copies (Figure 1F).

It is worth mentioning that a re-analysis of our suspension culture small RNA-seq study [34] yielded a 139 nt small nuclear RNA encoded within the Crick-strand of the rDNA in the Cas9-targeted region of the 25S rDNA, named herein SnRn25S (Figure S2A, B). The SnRn25S transcript was present in the nuclear fraction of the suspension culture and in the rosette, root, seedlings, and flowers tissues (Figure S2C, D). Within the mutated region of 25S rRNA, the two residues, G2620 and A2641 are likely to be methylated by at least four known C/D box snoRNAs, SnoR35, U31, SnoR27 and SnoR68Y [24] (Figure S2A). However, the cis-mutation of the rDNA regions did not cause inadvertent effects on the levels of these trans-regulatory snoRNAs (Figure S2E).

Genomic PCR and sequencing analysis with the mutation-specific forward and 3’-ETS-specific reverse oligos were used to deduce the chromosomal locations of the mutations through their distinguishable VAR1–4 polymorphisms (Figure 1B). The analysis resulted in bands unique to the *ploop KD* ± and *KD* -/- genotypes. Independent clones of the *KD* -/- product were sequenced (*n* = 33), which revealed that the mutations correlated with the VAR2, VAR3 and VAR4 polymorphic types (Figure 1G). This indicates that the mutations were predominantly associated with NOR4. Nevertheless, the absence of VAR1-linked copies to mutated alleles may not necessarily rule out disruption of NOR2 copies. The activity of Cas9 on presumably specific NOR could, in part, be ascribed to their higher order nucleolus-associated chromatin domain (NAD) configurations of specific NOR during plant development [35] In mammalian cells, a discernible link has been established between nucleosome acting as inhibitors of Cas9 activity and open chromatin serving as a determinant for Cas9 efficiency [36–38]. Taken together, localized chromain dependency and 35S constitutive promoter driven expression of Cas9 during vegetative stages of peak NOR4 activity can likely cause such selective NOR4 targeting. A recent study on Arabidopsis rDNA mutagenesis via Cas9 reported that the egg cell-specific targeting of rDNA copies using single guide with SpCas9 led to the loss of up to 20% of the rDNA copies with no obvious growth defects [39]. In another report, the targeting of the ITS2 regions of the repetitive rDNA copies using a single guide with the ubiquitin promoter-driven superior Cas9 (SaCas9) led to a high lethal rate among the transformants relative to the control gene targeting ADH1. Furthermore, the tissue-specific Cas9-based mutation of the ITS2 region has been used to eliminate specific organs, i.e. the lateral roots and floral organs [40]. Our study, on the other hand, employs the dual guide-based targeting of the rDNA using the 35S promoter-driven SpCas9 to induce fragment excisions without the loss of rDNA copies, thus explaining the variations in the induction of the double-strand breaks in the sex cells compared to the somatic cells, effects of variable chromatin configurations, and the variable potency of Cas9 derived from different species. Alternatively, the stable inheritance of phenotype in T2–4 alone could be due to high lethal rate among transformants overall as was also observed using SaCas9 with ubiquitin promoter [40], thus explaining the low probability of obtaining such stable defective phenotype-induced transformants.

### Mutation caused growth defects linked to a single locus

**Figure 2.**
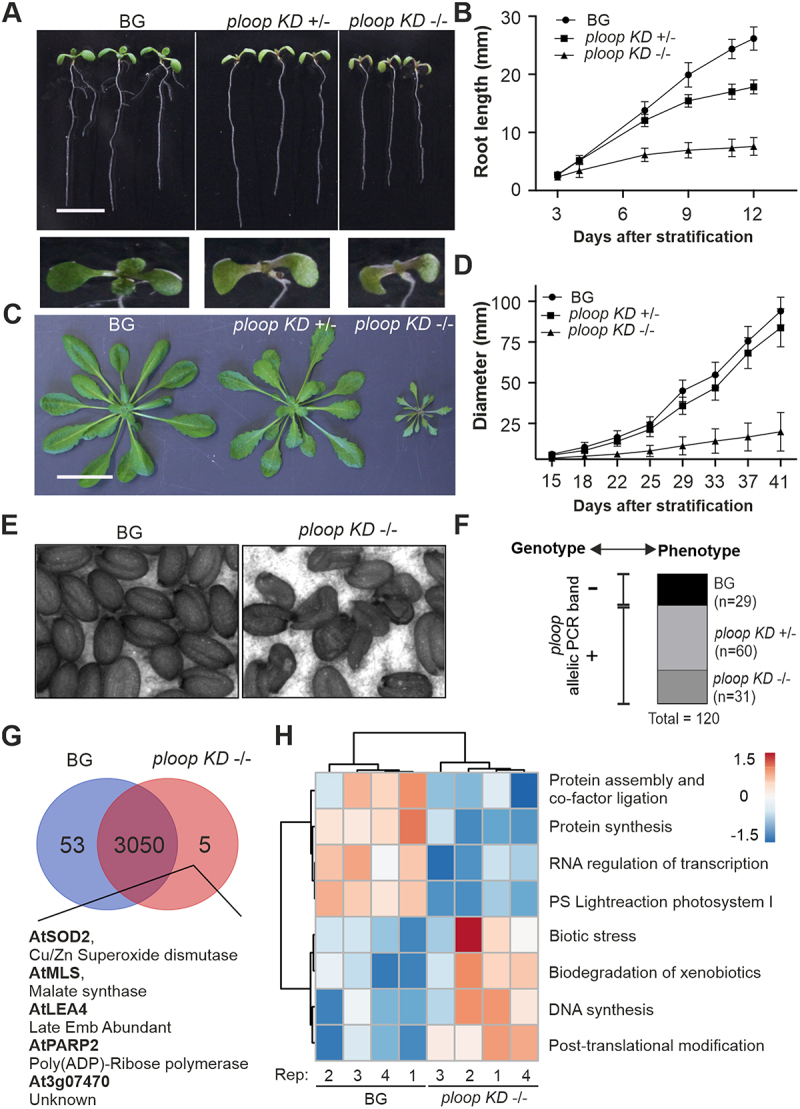
Mutation caused dosage-dependent growth defects linked to a single locus. (A) Seedling growth phenotypes of segregating heterozygous plants. Scale bar: 10 mm (B) root length variations analysed during early growth (*n* = 18, BG; *n* = 29, *ploop KD* ±; *n* = 16, *ploop KD* -/- vertically grown seedlings). (C) Rosette morphology of soil-grown mutants. Scale bar: 30 mm (D) rosette diameters of mutants (*n* = 18 replicates for each genotype). (E) Seed morphology of *ploop* KD -/- mutants. (F) Genotype-phenotype correlation of 120 randomised seedlings based on the phenotypical segregation as in figure 2A and further genomic PCR analysis of the *ploop* allelic band (+, present; -, absent) of all seedlings. (G) Venn analysis of the identified proteome based on 4 biological replicates of 10 DAS BG and *ploop KD* -/- seedlings. (H) Bicluster analysis of the abundance of varying levels of GO terms-based classified proteins between 4 replicates. Data in (B) and (D) are presented as means ± SD.

The mutants displayed dosage-dependent growth and developmental defects due to the large number of copies targeted. The seedlings of the three genotypes consistently displayed morphological distinctions. In comparison to the BG seedlings, the *ploop KD* ± seedlings lacked lateral root growth and exhibited delayed first-leaf emergence. The *ploop KD* -/- seedlings were, overall, smaller in size in comparison to both the BG and *ploop KD* ±, while being morphologically similar to the *ploop KD* ± in terms of the absence of lateral root growth and first leaf emergence (Figure 2A). The root growth rate up to 7 DAS was comparable between the BG and *ploop KD* ± seedlings, although in the *ploop KD* ± seedlings this was reduced by 8 mm at 12 DAS. The *ploop KD* -/- seedlings showed reduced root lengths of up to 7 mm at 7 DAS and 18 mm at 12 DAS in comparison to the BG (Figure 2B).

At later vegetative growth stages, the rosette morphology of the *ploop KD* ± plants reached the size of the BG plants, while the *ploop KD* -/- remained severely stunted in overall growth with a drastically reduced leaf size. The leaves emerging from the shoot apical meristem of the *ploop KD* ± were more serrated on the sides and possessed pointy tips in comparison to the BG plants. The leaves of the *ploop KD* -/- were sharply serrated, pointed at the tip and lacked any similarity to the BG plants (Figure 2C). The rosette diameter of the *ploop KD* -/- plants was almost ten times reduced in comparison to the BG (Figure 2D). Furthermore, most of the *ploop KD* -/- plants failed to set seeds, however, if seeds were formed, then they were found to be shrunken and deformed (Figure 2E).

The *ploop* mutation genotype and observed phenotype correlation was established by mutation-specific PCR on the back-crossed segregating progenies (Figure 2F). A large-scale (*n* = 120) selection of randomized seedlings phenotypically segregated as BG (29), *KD* ± (60) and *KD* -/- (31) plants at 12 DAS in a 1:2:1 Mendelian ratio (Figure S3A). In the genomic analysis, all BG plants lacked the mutation-specific band, while the respective *KD* ± and *KD* -/- plants were all typified by the presence of specific mutation allelic bands (Figure S3B). Thus, the mutated versions of the rDNA copies were located in NOR4 of chromosome 4, and the mutation (genotype) and phenotype were segregated together (Figures 1, 2).

Proteome analysis on the BG and *ploop KD* -/- seedlings (*n* = 4 biological replicates) was performed to define the molecular basis of the mutant morphology. The principal components 1 and 2, amounting to 62% of the total variance, resolved the two genotypic groups (Figure S4). Analysis showed that 3,050 proteins were commonly present in both genotypes, while 53 proteins were uniquely detected in the BG and only five proteins (AtSOD2, AtMLS, AtLEA4, AtPARP2, and At3g07470) in the *ploop KD* -/- seedlings (Figure 2G, Table 1). Biclustering analysis of both the BG and *KD* -/- genotypes, together with the cumulative functional ontological grouping, revealed that proteins involved in photosynthesis, protein synthesis and assembly, and RNA processing were markedly decreased in the mutants. In turn, the levels of proteins involved in DNA synthesis, post-translational modifications, biotic stress responses, xenobiotic degradation signalling proteins were increased in the *ploop KD* -/- mutants relative to the BG (Figure 2H, Figure S5, Dataset S1). The averaged abundance values across the replicates of the proteins regarding the GO terms, ribosome biogenesis and prokaryotic and eukaryotic origin ribosomal proteins showed that the RP and RBF stoichiometry was similar between both genotypes except for a few individual cases of variable abundance (Figure S6). In terms of the RBFs, out of 52 identifiable proteins, NOP10 (an H/ACA snoRNA complex protein), and IMP4 (a snoRNP complex protein); both belonging to RNA- based modified subcomplex proteins of early SSU processome complex were reduced in the mutant while, in turn, levels of pre-60S LSU complex BRIX domain containing protein, ARPF2 were increased in the mutant. Among RPs of prokaryotic origin, the levels of the plastid-specific 50S ribosomal protein 6 (PSRP6) levels were relatively higher in the mutant. With respect to the RPs of eukaryotic origin, at least 21 proteins were relatively decreased in the mutant among the 124 detected. Taken together, the mutation led to allele dosage-dependent defects in terms of the root and rosette developmental morphology, while the severe *KD* -/-phenotype was exemplified by global pleiotropic proteomic changes in the *KD* -/- seedlings with moderate effects on the ribosome biogenesis proteome.

**Table 1.** List of proteins distinctly absent in *ploop KD* -/- seedlings.

At Locus ID	Protein Description	Name^1^
AT5G17870	plastid-specific 50S ribosomal protein 6	AtPSRP6
AT2G42530	cold regulated 15b	AtCOR15B
AT5G58110	chaperone binding; ATPase activators	-
AT5G20935	Chloroplast NADH dehydrogenase assembly protein	AtCRR42
AT5G66550	Maf-like protein	-
AT3G10620	nudix hydrolase homolog 26	AtNUDX26
AT1G48610	AT hook motif-containing protein	-
AT4G16500	Cystatin/monellin superfamily protein	ATCYS4
AT5G15530	biotin carboxyl carrier protein 2	At BCCP2
AT3G52730	ubiquinol-cytochrome C reductase UQCRX/QCR9-like family protein	-
AT4G36430	Peroxidase superfamily protein	-
AT5G39210	chlororespiratory reduction 7	AtCRR7
AT3G10860	Cytochrome b-c1 complex, subunit 8 protein	-
AT1G77710	Ubiquitin-like, Ufm1	ATCCP2
AT1G01170	ozone-responsive-stress-like protein	-
AT5G20140	SOUL haem-binding family protein	AtHBP5
AT1G13730	Nuclear transport factor 2 (NTF2) family protein with RNA binding (RRM-RBD-RNP motifs) domain	-
AT2G05310	transmembrane protein	-
AT2G30695	bacterial trigger factor	-
AT3G05020	acyl carrier protein 1	AtACP1
AT4G39860	hematological/neurological-like protein	-
AT2G35390	Phosphoribosyltransferase family protein	-
AT5G26210	alfin-like 4	AtAL4
AT3G58990	isopropylmalate isomerase 1	AtIPM1
AT5G22580	Stress responsive A/B Barrel Domain	-
AT2G47580	spliceosomal protein U1A	AtU1A
AT1G32380	phosphoribosyl pyrophosphate (PRPP) synthase 2	ATPRS2
AT2G16850	plasma membrane intrinsic protein 2;8	AtPIP2, AtPIP3B
AT3G09150	phytochromobilin:ferredoxin oxidoreductase, chloroplast/phytochromobilin synthase (HY2)	ATHY2, ATGUN3
AT4G05520	EPS15 homology domain 2	ATEHD2
AT3G51820	UbiA prenyltransferase family protein	AtG4, AtPDE325
AT5G16810	Protein kinase superfamily protein	-
AT5G63140	purple acid phosphatase 29	ATPAP29
AT1G16410	cytochrome p450 79f1	ATBUS1, ATBUSHY1, ATSPS1, ATCYP79F1
AT4G36390	Methylthiotransferase	-
AT2G35795	Chaperone DnaJ-domain superfamily protein	AtPAM18–1
AT4G18970	GDSL-like Lipase/Acylhydrolase superfamily protein	AtGGL22
AT4G31780	monogalactosyl diacylglycerol synthase 1	AtMGD1, EMB2797
AT1G08530	chitinase-like protein	-
AT3G09250	Nuclear transport factor 2 (NTF2) family protein	-
AT3G25680	SLH domain protein	-
AT4G08360	KOW domain-containing protein	-
AT1G04970	lipid-binding serum glycoprotein family protein	AtLBR-1
AT3G51670	SEC14 cytosolic factor family protein/phosphoglyceride transfer family protein	AtPATL6
AT3G18240	Ribosomal protein S24/S35, mitochondrial	AtRPS24/35
AT3G20680	Domain of unknown function (DUF1995)	-
AT1G50140	P-loop containing nucleoside triphosphate hydrolases superfamily protein	-
AT1G06070	Basic-leucine zipper (bZIP) transcription factor family protein	AtBZIP69
AT4G20010	plastid transcriptionally active 9	ATOSB2, ATPTAC9
AT4G34020	Class I glutamine amidotransferase-like superfamily protein	ATDJ1C
AT3G13560	O-Glycosyl hydrolases family 17 protein	-
AT4G27180	kinesin 2	AtK2, ATKATB, ATKINESIN2
AT5G20360	Octicosapeptide/Phox/Bem1p (PB1) domain-containing protein/tetratricopeptide repeat (TPR)-containing protein	AtPHOX3

^1^The At Locus ID refers to the accession numbers, and names given according to the latest information on The Arabidopsis Information Resource (TAIR) in www.arabidopsis.org.

### Mutation of chromosome 4 copies induced compensation from the non-mutated chromosome 2 copies in mutants

**Figure 3.**
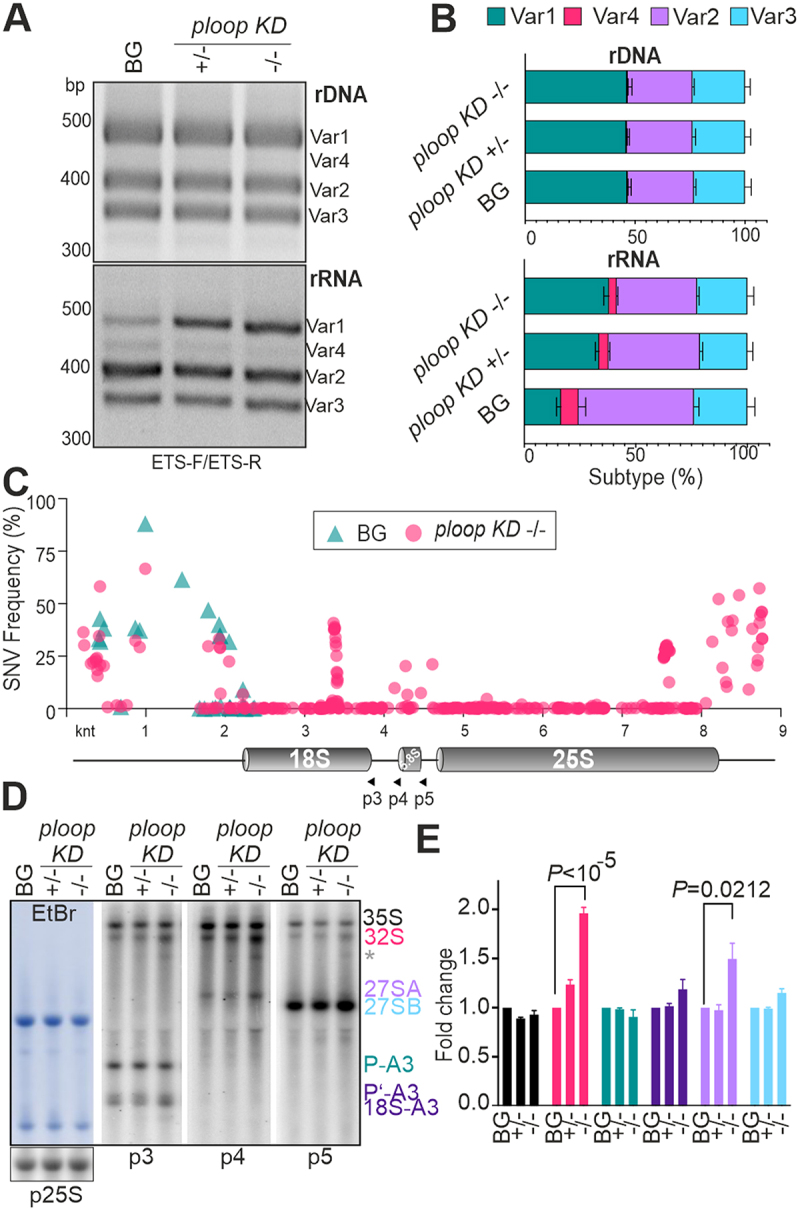
Mutation caused dosage compensation from the usually inactive NOR2 locus leading to other rRNA defects.

The VAR distribution pattern on the genomic DNA observed here was largely consistent with previous studies (Figure 3A,B – top panels) [7,8,39,41]. Across the genotypes, VAR1 encoded only on NOR2, was the dominant type followed by VAR2 and VAR3, with VAR4 encoded only on NOR4 being barely detectible. However, relative quantification of rDNA copies across three genotypes revealed slight proportional reduction of rDNA copies in both *KD* ± and *KD* -/- mutants relative to AtEF1Ba amplification (Figure S7A). Analysis of the cDNA yielded an expression dynamic of four variants during the early seedling growth stages in wild type, with VAR1 exhibiting the highest expression at 3 DAS and a subsequent decline (Figure S7B). Comparison of the three genotypes at 14 DAS yielded elevated VAR 1 RNA levels, significantly reduced VAR 4 and VAR 2 RNA levels and similar VAR 3 RNA levels in the *ploop KD* ± and *KD* -/- mutants compared to the BG (Figure 3A,B – bottom panels). Moreover, in the apex tissues of 35-days-old plants with the pointy leaf phenotype, VAR1 was still expressed in the *ploop KD* ± plants, but not in the BG (Figure S7C). This demonstrates a consistent VAR1 ectopic expression and, thus, an overall imbalance in the distribution of rRNA variants in the mutants when compared to the BG. This compensation mechanism is consistent with previous reports. On the one hand, mutants lacking histone deacetylase 6 (HDA6), an enzyme that reverses the acetylation of lysine residues at H3K14, H4K5 and H4K12, exhibited ectopic expression of VAR1 at the seedling and adult stages [8,42,43]. On the other hand, VAR1 dosage compensation is a feature of mutants possessing reduced copy numbers of ribosomal DNA in Arabidopsis [39]. Thus, it is likely that surveillance pathways exist in cells to monitor the transcriptional outcomes of rDNA repeats in both chromosomes to compensate for the production of defective ribosomes.

(A) (B) Electrophoresis (A) and quantification of PCR products (B) following amplification with the ETS-F and ETS-R oligos (Figure 1B) from genomic DNA (upper panels) and cDNA, reverse transcribed from total RNA (lower panels) of 14-days-old BG and *ploop KD* mutants. PCR products (VAR1-4) are marked on the right-hand side and the DNA marker (bp) on the left. Data in (B) are presented as means ± SD % of each subtype variant to the total % of rDNA or rRNA. (C) SNV analysis of the 35S pre-rRNA from the BG (cyan) and *ploop KD* -/- (magenta) seedlings denotes the frequency of each position relative to the reference rDNA [22]. (D) Northern hybridization analysis of the total RNA from the BG and the *ploop KD* mutants with complementary radioactively-labelled oligonucleotides (p3, p4 and p5 position indicated in panel C) binding to the immature regions of ITS1 and ITS2; the EtBr stained on the left-hand side shown as the loading control. Identified precursors are indicated on the right. The asterisk denotes an abnormal decay product as reported earlier [45]. (E) Quantification of each pre-rRNA precursor relative to the radiolabelled 25S rRNA signal according to the colour scheme in panel E (*n* = 4 biological replicates). *ploop KD* ± and -/- are simply indicated by ± and -/-, respectively. Data are shown as means ± SD fold change values after normalizing with radiolabelled 25S rRNA signal and BG the genotype. The P-values denote the statistical significance with the unpaired student t-test.

In support of an altered rRNA usage, total RNA-seq analysis of the 14 DAS BG and *ploop KD* -/- mutant seedlings yielded a SNV frequency at the 3’-ETS region which was up to 60% higher in the mutants, while the 5’-ETS-related SNV frequencies also varied between the BG and *KD* -/- genotypes. More importantly, a polymorphic island associated with the 3’ region of 18S and 5S was also revealed in the RNA-seq analysis in the mutants, but not in the BG (Figure 3C, Dataset S2). The net lack- and ectopic expression of polymorphic rRNAs may result in overall imbalance of specialized ribosomes in the mutants since the coordinated expression of rRNA copies containing variable SNVs has been shown to be a characteristic source of tissue-specific ribosomes [22].

To explore changes in the ribosome biogenesis enforced by the rRNA variant imbalance, the levels of high and low molecular weight pre-rRNAs in the BG, *ploop KD* ± and *KD* -/- mutants were probed by northern hybridization (Figure 3D). The analysis revealed steady-state levels of seven high molecular weight pre-rRNAs (Figure S7D). The radioactivity of the bands was quantified and normalized to the levels of radio-labelled 25S rRNA (Figure 3E), this yielded a comparable level of the primary transcript 35S across all genotypes. The levels of the 32S diagnostic for the minor pathway [44] was two-fold higher (*P* < 10^−5^) in the *KD* -/- mutant than in the BG, while the P-A3 levels, a precursor specific for the major pathway, was comparable in all genotypes. The levels of precursors downstream of 32S, i.e. 18S-A3, 27SA (*P* = 0.0212) and 27SB, followed similar trends to 32S, but not to such an extent as 32S. Besides the canonical pre-rRNAs, an abnormal decay product below 32S could be detected in the *ploop KD* -/- mutant across three blots (asterisk, Figure 3D). A similar precursor decay product has been implicated as byproduct of stalled 60S subunit upon *rpl17* mutation in mammalian cells, suggesting a role for the core ribosomal components in pre-rRNA turnover through degradation of synthesized precursors [45]. Furthermore, no obvious changes in the levels of the 5’−5.8S, 6S and 7S LMW precursors were observed (Figure S7E). While any of the factors controlling the minor pathway maturation may be defective in the *ploop KD* -/- mutant, other ectopically expressed VAR1 pre-rRNAs may also contribute to such a pathway-specific breakdown. It has been reported earlier that RBFs exhibit tissue-specific expression [17]. Due to variations in pre-rRNA types during development (Figure S7B) and in different tissues, the ectopic expression of VAR1 could cause a specific defect in the minor maturation pathway by the lack of necessary tissue-specific or sequence-specific RBFs.

### Mutation carrying rRnas are not incorporated into translating polysomes

**Figure 4.**
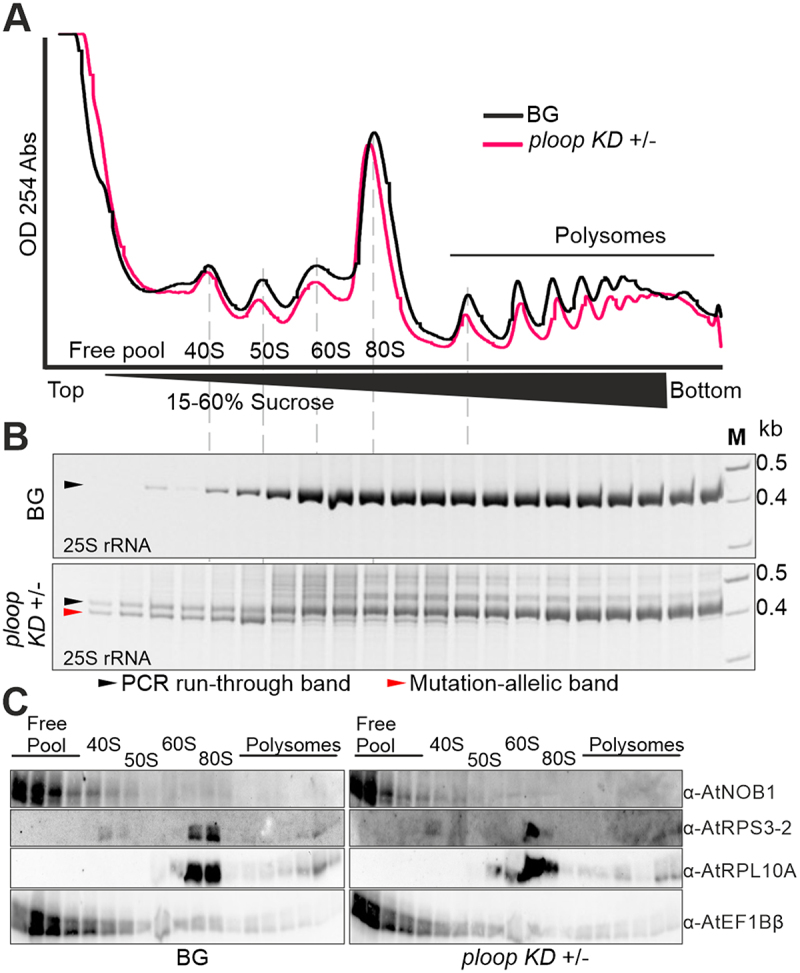
Mutated copies are not incorporated into the translating polysomes. (A) Absorbance profiles at 254 nm of the floral tissues from BG (black) and *ploop KD* ± (magenta) seedlings plotted with time of detection from top to bottom following sucrose-density gradient centrifugation. (B) The RNA from the corresponding fractions in panel a for both genotypes was purified, reverse transcribed with the 25S rRNA-specific oligo and the PCR products with the mutation-specific forward oligo and 25S-specific reverse oligo resolved by 12% native PAGE gels. The black arrow denotes the PCR-run through product and the magenta arrow indicates the mutated rRNA product. (C) The proteins from the fractions in panel a of both genotypes were resolved on 10% SDS-PAGE and blotted with the indicated antibodies shown on the right-hand side.

To deduce the distribution of the mutated rRNA copies on the corresponding ribosomal pre-subunits, maturing pre-ribosome and polysome populations of the BG and *ploop KD* ± floral tissues were fractionated by density-gradient centrifugation. The absorbance profile of the lysates revealed a separation of peaks consisting of a free pool of proteins and RNA, 40S, prokaryotic 50S, 60S, 80S monosomes and polysomes (Figure 4A). RNA and protein were purified from the density fractions. RT-PCR with a 25S-specific oligo and the mutation-specific forward primer was conducted to detect the distribution of mutated rRNA copies (Figure 4B). The BG and *ploop KD* ± tissues contained wild-type 25S, as judged from the PCR product; this was present in the 60S fraction and all subsequent polysome fractions. In the case of the *ploop KD* ± tissues, a clear band was detectable below the wild-type band corresponding to the deletion of ~ 25 bp and, thus, indicating the mutated-allele-specific product. The mutated product was mainly detected in the free pool fractions but also within the 50S-sized fraction. A weaker fraction of mutant rRNA could still be detected in other fractions from the 60S to the lower order polysomes, but was not present in the higher order polysome fractions. The identity of the complexes was confirmed by immunoblotting with antibodies against the ribosome biogenesis factor NOB1, a small subunit ribosomal protein (RPS3–2) and a large subunit ribosomal protein (RPL10A), as well as a translation elongation factor (EF1Bβ) (Figure 4C). This analysis revealed that NOB1 and EF1Bβ are mostly concentrated in the free pool fractions of both genotypes. RPS3–2 is present in the 40S and 80S fractions, while RPL10A was predominantly detected in the 80S, but also in the polysomes and 60S fractions. The analysis of the silique tissues yielded comparable results to those of the flower tissues (Figure S8).

It is likely that the mutational copies that are detected within the monosome fractions correspond to the copies being detected from the initial pre-90S complex. Based on the intensity of the band arising within the fractions of two different tissues, the floral and silique, we observed that the detectable mutated copies were present more in the maturing fractions than in the 80S fractions (Figure 4B, Figure S8B). This, in part, indicates that it is less likely or, indeed, unlikely that active monosomes or polysomes would contain the mutated P-loop fragment. Moreover, the P-loop region of the eukaryotic ribosome region appears to interact with the N-terminal region of the RPL29 protein [33]. In addition, the P-loop region lies in close proximity to RPL10 although its interaction with the P-loop has not been verified. Given that the P-loop region possesses a minimum of 3 active residues, based on their interaction potential with other proteins [46], it is plausible that the binding of these two proteins to the ribosomal RNA may not be facilitated due to the direct result of the mutation.

### Mutants displayed elevated autophagic flux

**Figure 5.**
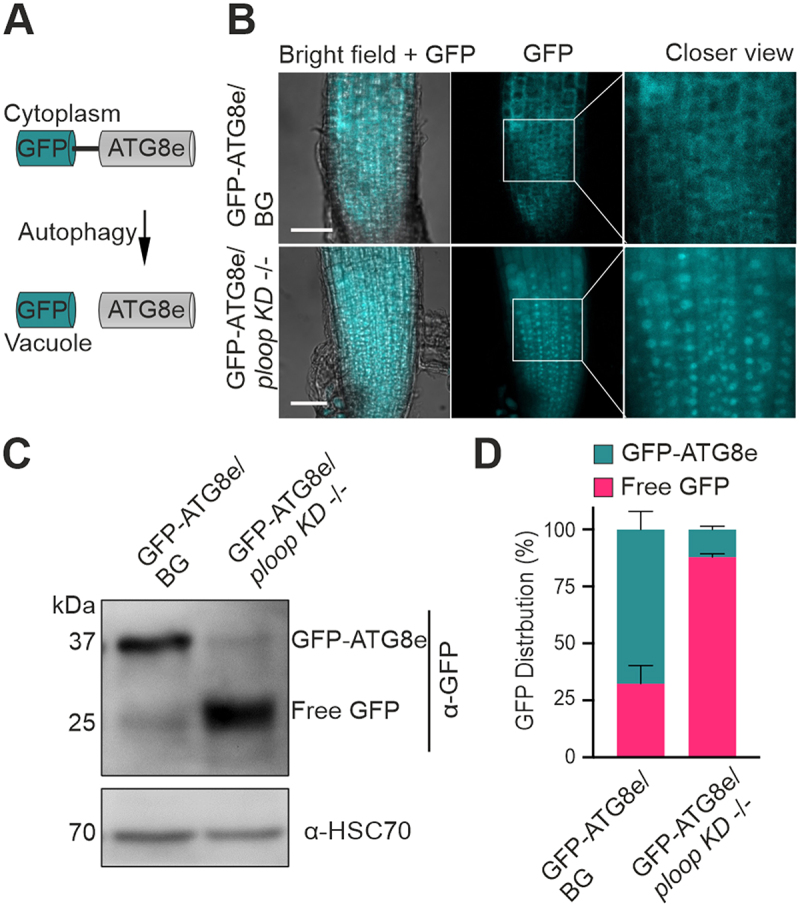
Mutants display elevated autophagic flux. (A) Schematics of the GFP-ATG8e fusion protein localisation under routine growth and autophagic conditions. (B) Localisation of the GFP signals from the GFP-ATG8e transgenic fusion proteins in the BG (top) and *ploop KD* -/- (bottom) seedlings. Scale bars: 50 µm (C) immunoblotting analysis with the anti-GFP antibodies of the GFP-ATG8e levels in the BG and *ploop KD* -/- seedlings. Anti-HSC70 blotting served as the loading control. (D) Quantification of the full length GFP-ATG8e and free GFP signals from panel C represented in relation to the total levels after normalisation to the HSC70 loading control (*n* = 3 biological replicates). Data are presented as means ± SD % of each GFP variant to the total GFP signal intensity after normalizing with the HSC70 loading control signals.

The absence of mutated rRNAs in the polysomes prompted us to postulate that defective immature or non-functional matured ribosomes may be degraded and, thus, resulting in an enhanced autophagic rate in the *ploop KD* -/- genotype. Consequently, the autophagic levels in the mutant were analysed by expressing a fluorescent reporter transgene, GFP-ATG8e [47], which is a structural component of the autophagosomes [48,49]. The full-length protein is localized and diffusely distributed in the cytoplasm under control conditions [50]. This pattern was observed for the BG in the root tip region (Figure 5A,B – top panels). Furthermore, elevated levels of autophagy mobilize the fusion protein to the vacuoles [50], which was observed in cells of the *ploop KD* -/- genotype root tips (Figure 5A,B – bottom panels).

Within the vacuoles, the protein is cleaved, however, the free N-terminal GFP residue remains resistant to vacuolar hydrolases and proteases. Thus, free GFP formation is an indication of the turnover of these proteins to the autophagosomes (Figure 5A). In the GFP immunoblotting analysis of the BG genotype, the full-length GFP-ATG8e signal was dominant, accompanied by a slight intensity of free GFP signal. In contrast, the full-length GFP-ATG8e form was barely detectable in the *ploop KD* -/- seedlings with a predominant signal obtained for the free GFP (Figure 5C). The quantification of the observed signals after normalizing with the HSC70 levels indicated that, in comparison to the BG genotype, the ratio of free GFP was at least three-fold higher while the fused GFP-ATG8e ratio was at least seven-fold lower in the *ploop KD* -/- seedlings (Figure 5D).

The formation and persistence of immature ribosomal RNAs would likely trigger recycling mechanisms in the cell. This assumption is consistent with our analysis which shows that, indeed, autophagic turnover rates are high even under control growth conditions. This constitutive autophagy may pose additional stand-alone challenges for the growing cell. The chemical and molecular components of these autophagosomes would shed more light on the ribophagy mechanisms and the conditions in which they are triggered. If the elevated autophagy levels are, indeed, due to the defective ribosomes, it would indicate that the ATG8e orthologue would share the ribophagy cargos as well, rendering them even more versatile.

## Materials and methods

### Growth of plants and phenotypic analysis

The growth of Arabidopsis plants on media has been described previously [44]. In brief, the seeds were surface sterilized in two steps: 0.1% (v/v) Triton X-100 for 1 h followed by 70% ethanol for 1 min. The excess ethanol was removed by 5X washing with sterile distilled water. The seeds were spread on MS (Murashige-Skoog) medium (Duchefa Biochemie) supplemented with 1% (w/v) sucrose and 0.8% (w/v) phytoagar and stratified for 3 d at 4°C. The plates were maintained in long-day growth conditions of 16 h/8 h light-dark cycle at 22°C. Plants in soil were maintained using similar growth conditions in a walk-in growth chamber.

The phenotypic analysis was performed by photographing seedlings grown vertically on MS containing 1% (w/v) sucrose and adult plants grown in soil. A Canon EOS 7D camera was used for photography, while the series of images were processed in ImageJ (NIH, USA) for the root length and rosette diameter measurements.

### CRISPR mutagenesis

For CRISPR-mutagenesis of the target genomic region, two guide RNAs were selected using CRISPR-P (http://crispr.hzau.edu.cn/CRISPR2/) for the target region and the constructs were created using the Golden Gate vector system, as described earlier [51]. The guide sequence-containing PCR forward oligonucleotides, CRISPR-g1F and CRISPR-g2F, were used along with the reverse oligo CRISPR_R (Table S1) to create g1RNA-sgRNA and g2RNA-sgRNA products using plasmid #46968 (Addgene) as the template DNA. The resulting PCR products of the individual guides were combined with the AtU6.26 promoter (Source: pICSL01009) and assembled further into Level 1 vectors (pICH47732 for guide 1; pICH47742 for guide 2). The resulting Level 1 clones were subsequently combined with an end-linker (pICH41744) in order to mobilize into a binary expression vector (pICSL002208) using level 2 reaction. The resulting expression clone, containing 35S:SpCas9 and 35S:nptII for Cas9 expression and kanamycin resistance, respectively, was transformed into the GV3101 *Agrobacterium* strain for the floral dipping-based transformation of Col-0 plants [52]. The resulting T1 seeds were selected on MS media containing 50 μg/mL of kanamycin (K0126, Duchefa Biochemie).

### Heteroduplex PCR, cloning and sequencing

The mutant plants were identified by heteroduplex PCR [53] using a genomic DNA template. Briefly, following the crude or pure isolation of genomic DNA, PCR was conducted using mutation site flanking oligos (Flank-F/R); the amplified products were denatured for 10 min at 95°C and cooled down at 1°C min^−1^ using the cycler programming until 25°C was reached. The products were resolved on native 15% polyacrylamide containing gels in 1X TBE (90 mM Tris, 90 mM Boric acid, 2 mM EDTA pH 8.0) at 150 V and stained with ethidium bromide for 10 min. The higher order differential heteroduplex-containing F1 plants, compared to the wild type were propagated and an independent T2 line carrying the mutation and lacking the Cas9 expression cassette was identified by PCR using Cas9-F/R oligos. For quantification of intact and mutated copies in mutants (Figure 1D,E), the EtBr stained gels were processed in ImageJ (NIH, USA). The intact homoduplex and mutated heteroduplex band intensities were measured for each lane and these density values were converted to percentage values with respect to total rDNA in each genotype and plotted using Graphpad Prism 9 (www.graphpad.com).

The PCR products in this study were cloned into the TA vector, pGEM-T Easy (Promega, USA). Insert-containing plasmids were purified from white colonies and subsequntly sequenced with either the M13(−20)-F or M13(−20)-R sequencing primers (Eurofins Genomics, Germany).

### Proteomic analysis

For proteomic analysis, Mendelian-segregating *ploop KD* ± plant-derived seeds were grown on MS containing 1% (w/v) sucrose for 10d. The seedlings were phenotypically scored to background and homozygous-looking seedlings and were then ground to fine powder in liquid nitrogen. The proteins were extracted, pre-fractionated (1D SDS-PAGE), trypsin digested and desalted (using a C18 spec plate) using the shotgun proteomics approach according to previously described methods [54,55]. Prior to mass spectrometric measurement, the tryptic peptide pellets were dissolved in 4% (v/v) acetonitrile, 0.1% (v/v) formic acid. One microgram of peptides was separated by an online reversed-phase (RP) HPLC (Thermo Scientific Dionex Ultimate 3000 RSLC nano-LC system) connected to a benchtop Quadrupole Orbitrap (Q-Exactive Plus) (QE Plus) mass spectrometer (Thermo Fisher Scientific). The online separation was performed on an Easy-Spray analytical column (PepMap RSLC C18, 2 μm, 100 Å, 75 μm i.d. ×50 cm, Thermo Fisher Scientific) with an integrated emitter. Separation was achieved with a 90 min gradient from 98% solution A (0.1% formic acid in high purity water(MilliQ)) and 2% solution B (90% ACN and 0.1% formic acid) at 0 min to 40% solution B (90% ACN and 0.1% formic acid) at 90 min with a flow rate of 300 nL min^−1^. nESI-MS/MS measurements were performed with the following settings: full scan range 350^−1^, 800 m/z resolution 120,000 max. 20 MS2 scans (activation type CID), repeat count 1, repeat duration 30 sec, exclusion list size 500, exclusion duration 30 sec, charge state screening enabled with the rejection of unassigned and + 1 charge states, minimum signal threshold 500.

Raw data were searched with the SEQUEST algorithm present in Proteome Discoverer version 1.3 (Thermo Fisher, Germany) as described previously [55,56]. For the identification of proteins, protein FASTA from the TAIR database was used. Peptides were matched against these databases plus decoys, considering a significant hit when the peptide confidence was high, which is equivalent to a false discovery rate (FDR) of 1%, and the Xcorr threshold was established at 1 per charge (2 for + 2 ions 3 for + 3 ions, etc). The variable modifications were set to acetylation of the N-terminus and methionine oxidation, with a mass tolerance of 10 ppm for the parent ion and 0.8 Da for the fragment ion. The number of missed and non-specific cleavages permitted was two. There were no fixed modifications, as dynamic modifications were used. All the MS/MS spectra of the identified proteins and their meta-information were deposited on PRIDE repository (Accession no. PXD035623). The identified proteins were quantified based on the total ion count, followed by an NSAF normalization strategy [57].

### RNA purification, sequencing, and SNV analysis

RNA purification in this study was carried out using E.Z.N.A Plant RNA kit according to the manufacturer’s instructions. For RNA-seq analysis, the RNAs were purified from 16-days-old BG and *ploop KD* -/- seedlings and were initially analysed for RNA integrity and quality using the Bioanalyzer. RNA-Seq library preparation was performed on total RNA without rRNA depletion step while subsequent cDNA sequencing was performed using the NovaSeq 6000 by the Novogene sequencing facility. Paired-end reads of a length of 2 × 150bp were mapped specifically against the annotated precursor rDNA repeat of *A. thaliana* [22] using Bowtie 2 [58] tools with default parameter settings. As a result, 35 million reads were properly mapped to the rRNA in the BG and *ploop KD -/-* from a sum of 63 and 60 million reads, respectively. The output was sorted using Samtools [59]. SNV calling was performed using LoFreq [60] and assigned according to previous annotations [22]. Besides the default parameter settings, standard input-output paths and parallelization options, an additional parameter for LoFreq was performed using the – call-indels option to define possible insertions and deletions.

### Semi-quantitative, quantitative PCR and RT-PCR

For 3’-ETS RT reactions, 100 ng of RNA (pre-treated with DNase I (Roche Diagnostics GmbH, Germany)) were reverse transcribed using the ETS-R oligo in a 20 μL reaction using Super Script III Reverse Transcriptase (Thermo Fisher, USA) according to the manufacturer’s instructions. The resulting cDNA was adjusted to a volume of 100 μL with water and the variable ETS region was amplified using the ETS-F and ETS-R oligos using 5 μL cDNA template in a 25 μL reaction with Accustart II PCR ToughMix (QuantaBio, USA) using the recommended amplifying conditions but with a reduced extension period of 7 sec and a final extension of 2 min. For genomic DNA PCR, 5 ng of DNA template were used in the same Accustart II PCR Toughmix reaction. For genomic DNA quantitative PCR, 5 ng of template DNA was used with SYBR mix PowerUp (Thermo Fisher, Germany) and 0.03 μM of each primer set separately to amplify 5.8S rDNA and AtEF1Ba (Table S1) in StepOne Plus Real Time PCR cycler (Applied Biosystems, US). Relative quantification was performed according to 2^−ΔΔCt^ method [61].

### Northern blotting and hybridisation

Northern hybridization was carried out as described previously [62]. In the case of HMW RNA, 10 μg of total RNA were electrophoresed on a large 1.2% (w/v) agarose gel tank in 1X BPTE buffer (10 mM PIPES, 30 mM Bis-Tris, 1 mM EDTA pH 6.5) and vacuum blotted onto a positively charged Hybond N+ membrane (GE Healthcare, UK). In the case of LMW RNA, 5 μg of total RNA were electrophoresed on a 7 M urea-containing denaturing 6% or 10% PAGE gel in 1X TBE. The RNA was subsequently electroblotted onto Hybond N+ membranes. Both the HMW and LMW RNAs on the membranes were UV cross-linked using auto cross-link settings (UV Stratalinker 2400, Stratagene, USA). The membranes were hybridized overnight at 37°C using complementary oligo probes that were 5’ end-labelled with [γ-^3 2^ P] ATP (SRP-201, Hartmann Analytic, Germany) using T4 polynucleotide kinase (Thermo Fisher Scientific, USA). The washed membranes were exposed to a storage phosphor-imaging screen and autoradiography was recorded using the Typhoon Scanner 9400 (GE Healthcare, USA). For quantification of blots in Figure 3D, the phosphor-images were processed in ImageJ (NIH, USA) to generate lane values based on pixel intensity. The precursor rRNA values were first normalized to unchanged mature 25S signal and subsequently fold change values of mutants were reported with respect to background genotype set to 1. The graphs were plotted using GraphPad Prism 9 version (www.graphpad.com).

### Sucrose-density gradient fractionation and analysis

Polysome fractionation was performed as described previously [63]. Briefly, 300 mg of fresh weight floral tissues from background and heterozygous plants were ground in liquid nitrogen and dissolved in 400 μL of REB buffer. The tissues were allowed to thaw on ice with intermittent mixing. The lysate was end-to-end rotated at 4°C for 20 min and centrifuged at 20,000 g for 2 min. Equal volumes (350 μL) of the supernatants, having OD595 nm equivalents of 0.7, were loaded onto a 13 mL capacity polypropylene tube containing a 15–60% linear sucrose gradient. The tubes were carefully loaded onto a swinging bucket TST41.14 rotor and centrifuged at 4°C for 18 h and 98,900 g acceleration in a SORVALL Discovery 90SE ultracentrifuge (Kendro Laboratory Products, USA). The samples were fractionated using a gradient pump fractionator (Teledyne ISCO, USA) at a 15 sec and 60% pump speed setting. From the 500 μL of each collected fraction, 300 μL and 175 μL were used for RNA and protein purifications, respectively [44].

For the fractionated RNA-based RT-PCR, the purified RNA was treated with DNase and reverse transcribed in a 10 μL reaction using the 25SR oligo according to the RevertAid RT kit (ThermoFisher Scientific, USA). The cDNA templates were used in a PCR using the Mut-F and 25SR oligos and the resulting products were resolved on 12% native polyacrylamide gels.

The protein samples were resolved on large-sized (18×16 mm) 10% SDS-containing polyacrylamide (37.5:1 acrylamide: bisacrylaamide) gels using standard dual cooled vertical electrophoresis unit, SE600 (Hoefer, Germany) and were subsequently electroblotted onto a nitrocellulose membrane; the membranes were then blocked using 5% (w/v) non-fat milk powder containing 1 X PBS. The membranes were probed with antibodies as described earlier [44].

### Autophagic analysis

The transgenic autophagic marker lines expressing GFP-ATG8e pollen were crossed with the *ploop KD* ± mutant ovules. From the resulting F1 plants, phenotypically heterozygous for the *ploop* allele, an F2 line homozygous for GFP-ATG8e and heterozygous for the *ploop* allele was propagated on solid MS containing 1% (w/v) sucrose media. Seedlings that were 5-days-old were mounted onto a microscopic slide hydrated under the cover slip. GFP fluorescence was observed using an excitation wavelength of 488 nm through an argon laser and the resulting emissions were recorded between 505 and 525 nm using an LSM 780 Confocal laser scanning microscope (Carl Zeiss Microscopy, Germany).

For western blotting, 20 mg of 12 DAS seedlings were ground directly in 100 μL of 1X SDS-Sample buffer (50 mM Tris-Cl pH 6.8, 2% (w/v) SDS, 0.1% (w/v) bromophenol blue, 10% (w/v) glycerol, 100 mM DTT) using a plastic pestle and denatured at 95°C for 10 min. The supernatant was clarified by centrifugation before loading onto a 10% SDS-PAGE gel. The proteins were blotted onto a PVDF membrane and the GFP levels were probed with mouse monoclonal anti-GFP antibodies (#11814460001, Roche). The blots were stripped and re-probed with the anti-HSC70/HSP73, SPA-817 antibody (Enzo LifeSciences, Lörrach Germany) to ensure equal loading.

## Supplementary Material

Supplemental Material

Dataset S1.xlsx

Dataset S2.xlsx

## Data Availability

The RNA-seq data presented in this study are deposited in GEO repository under accession no. GSE213764 and the proteome data are deposited in PRIDE repository with accession no. PXD035623.
