# Supplementary material for: Low dose ribosomal DNA P-loop mutation affects development and enforces autophagy in Arabidopsis
Source: RNA Biol. 2023 Dec 29;21(1):1–15. doi: 10.1080/15476286.2023.2298532 (PMC10761087; doi:10.1080/15476286.2023.2298532)
Supplement: Supplemental Material [file KRNB_A_2298532_SM7061.docx]

***Supplement to***

**Low dose ribosomal DNA P-loop mutation affects development and enforces autophagy in Arabidopsis**

Thiruvenkadam Shanmugam^a^, Palak Chaturvedi^b^, Deniz Streit^a^, Arindam Ghatak^b^, Thorsten Bergelt^a^, Stefan Simm^a,c^, Wolfram Weckwerth^b,d^, Enrico Schleiff^a,e *^

^a^Institute for Molecular Biosciences & Buchmann Institute for Molecular Life Sciences; Goethe University Frankfurt, Max von Laue Str. 9, D-60438 Frankfurt am Main, Germany; ^b^Molecular Systems Biology (MOSYS), Department of Functional and Evolutionary Ecology, University of Vienna, Djerassiplatz 1, 1030 Vienna, Austria; ^c^Institute of Bioinformatics, University Medicine Greifswald, D-17475 Greifswald, Germany; ^d^Vienna Metabolomics Center (VIME), University of Vienna, Djerassiplatz 1, 1030 Vienna, Austria; ^e^Frankfurt Institute for Advanced Studies, Ruth-Moufang-Str. 1, D-60438 Frankfurt am Main, Germany.

Table S1. List of oligonucleotides used in this study.

Figure S1. Screening and identification of CRISPR-induced mutations.

Figure S2. Small RNA analyses associated with the mutated P-loop region.

Figure S3. Genotype-phenotype correlation studies in back-crossed progenies.

Figure S4. Principal component analyses of analyzed proteomes.

Figure S5. Bicluster analyses of analyzed proteomes.

Figure S6. Ribosome stoichiometry changes in BG and *ploop KD* -/- mutants.

Figure S7. Complementary analyses of BG and *ploop KD* -/- plants.

Figure S8. Analysis of *ploop* mutated rRNA distribution in silique tissues.

Dataset S1. Proteome measurement analysis of BG and *ploop KD* -/- seedlings

Dataset S2. SNP analysis on pre-rRNA of BG and *ploop KD* -/- genotypes.

| **Table S1.** List of oligonucleotides used in this study. Given is the name, the sequence of oligonucleotide in 5' to 3' fashion and its usage. | | |
| --- | --- | --- |
| CRISPR-g1F | TGTGGTCTCAATTGGTGGGGAGTTTGGCTGGGGGTTTTAGAGCTAGAAATAGCAAG | CRISPR construct generation, detection and validation |
| CRISPR_g2F | TGTGGTCTCAATTGCATCTGTTAAAAGATAACGCGTTTTAGAGCTAGAAATAGCAAG |  |
| CRISPR_R | TGTGGTCTCAAGCGACAAAAAAGCACCGACTCG |  |
| CRISPR_seq | GAACCCTGTGGTTGGC ATGCACATAC |  |
| Flank-F | CAACCCCTGTTTTTGGTCCCAAG |  |
| Flank-R | GATTTCTGTTCTCGTTGAGC |  |
| Cas9-F | CTTCGACCTGGCCGAAGATG |  |
| Cas9-R | CGTATTTGACCTTGGTGAGC |  |
|  |  |  |
| SnRn25S | GTTTGGCTGGGGCGGCACATCTGTT | Northern Hybridization |
| SnoR35 | TGTCAGACGGTTCAGGGAGGACGTTTACTTCTTC |  |
| U31a | TTTGAGAGAATCAGACAAAAATAGTCAATCACCATG |  |
| SnoR27 | ATTGGATCTCAGGTTTTTCATGATTTGTCTTC |  |
| SnoR68Y | GCAGACACTAAACAGAAAACGCTGAGATCTG |  |
| U3.5 | CCAGGGTAAAAGGCCTGTCTCT |  |
| p3 | GGTCGTTCTGTTTTGGACAGGTATC |  |
| p4 | CGTTTTAGACTTCAGTTCGCAG |  |
| p5 | GGATGGTGAGGGACGACGATTTGTG |  |
| p25S | CGCGACGCGGGCATCAGTAGGG |  |
| 7SL | ACTGGGCAGCCCAGAAACATGC |  |
|  |  |  |
| Mut-F | GTCAGGTGGGGAGTTTGGCTAC | Mutation targeting PCR |
| 25S-R | CGCGACGCGGGCATCAGTAGGG |  |
| ETS-F | GACAGACTTGTCCAAAACGCCCAC | 3'ETS-specific PCR |
| ETS-R | CCTGGTCGAGGAATCCTGGACGATT |  |
| M13(-20)-F | TGTAAAACGACGGCCAGT | pGEM-T sequencing |
| M13(-20)-R | CAGGAAACAGCTATGAC |  |
|  |  |  |
| 5.8S-F | CGACTCTCGGCAACGGATAT | gDNA-templated quantitative PCR |
| 5.8S-R | TTGTGACACCCAGGCAGACG |  |
| EF1Ba-F | ACTTGTACCAGTTGGTTATGGG |  |
| EF1Ba-R | CTGGATGTACTCGTTCTTAGGC |  |

**
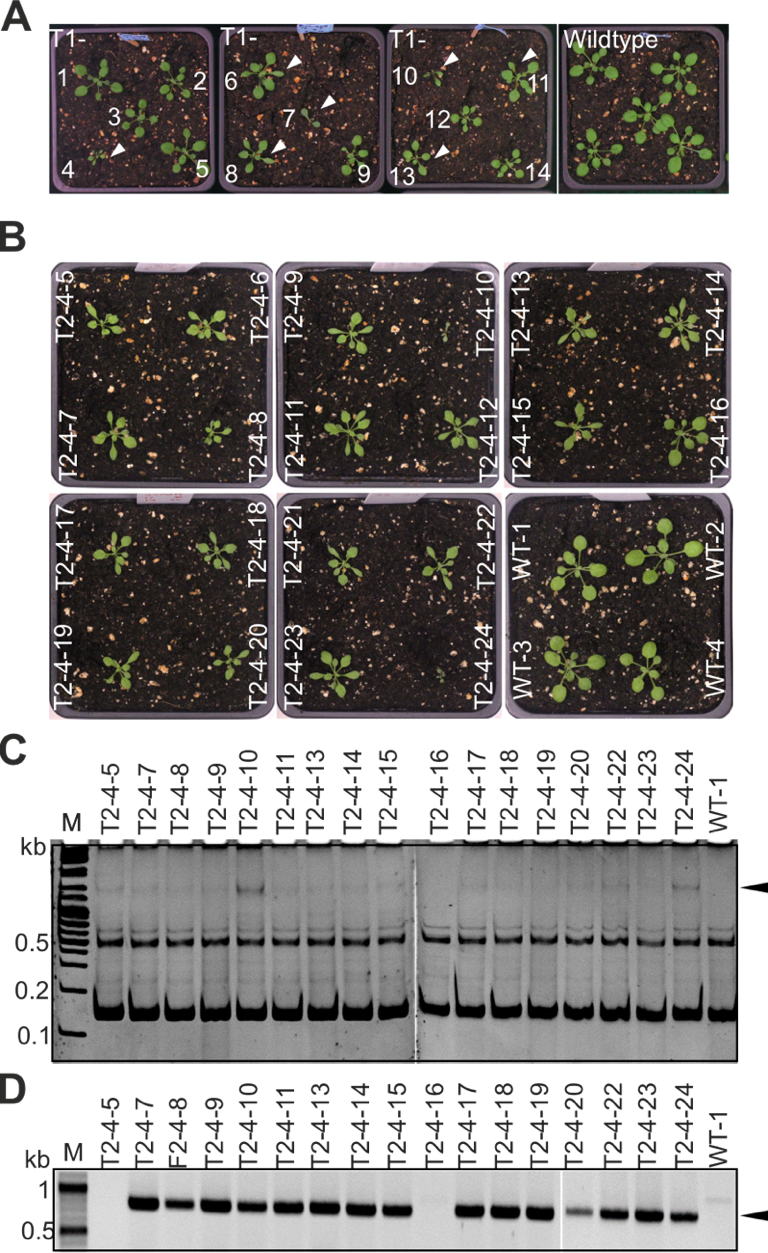
**

**Figure S1.** Screening and identification of CRISPR-induced mutations. (A) 23d old T1 plants (#1-#14) transplanted after growing in selection MS media containing kanamycin. White arrowheads denote the defective phenotypes (B) T2 plants raised from #T1-4 from non-selective media selected for the heritable pointy-leaf phenotypes (C) Heteroduplex PCR conducted on genomic DNA of indicated plants using oligonucleotides (Flank-F/R) to detect the mutation. Note the absence of heteroduplex band on F2-4-16 and its WT-like phenotype on panel B. In this analysis with phenotype displaying plants alongside wild type, the mutation containing plants were distinguishable by specific formation of heteroduplex loopy products through re-annealing of mutated and wild-type copies resulting in higher-order mobility under native PAGE conditions. (D) The same plants were used to test the absence of Cas9 expression cassette using specific oligonucleotides (Cas9-F/R) with genomic DNA PCR.


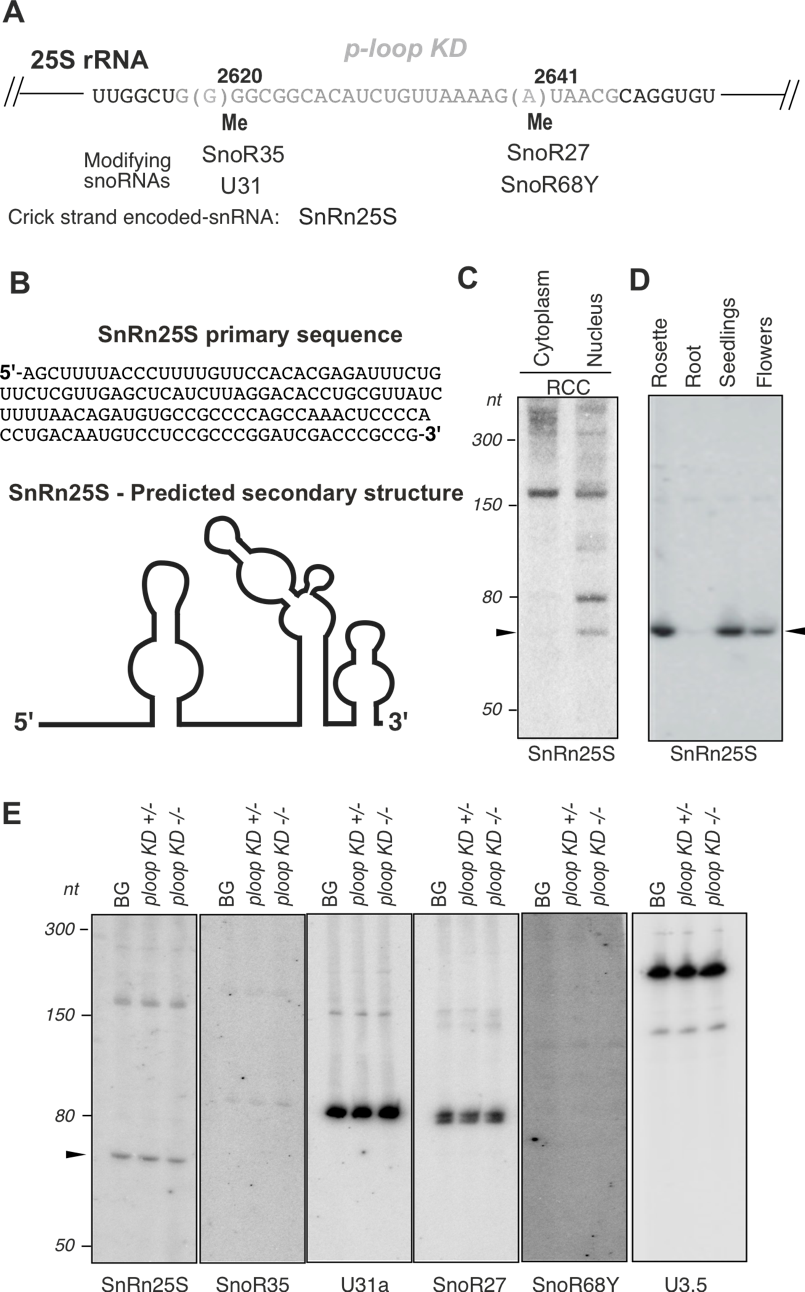


**Figure S2.** Small RNA analyses associated with the mutated P-loop region. (A) Small RNAs that are cis-derived and trans-acting in the mutated P-loop region of rDNA/rRNA (B) Primary sequence and secondary structure of the SnRn25S as predicted by RNAstructure (https://rna.urmc.rochester.edu) (C) Northern hybridization analysis of SnRn25S expression in fractionated cytoplasmic and nuclear extract of root cell cultures (RCC) with RNA marker (nt) shown on the left led to nuclear fraction specific ~80 nt transcript. (D) Northern analysis of SnRn25S expression in plant tissues; rosette, root, seedlings, and flowers. (E) Northern analysis of expression levels of SnRn25S and snoRNAs targeting the mutagenized region in BG and *ploop KD* mutants. While the U31a and SnoR27 are strongly expressed relative to weakly expressed SnoR35 and SnoR68Y, there were no obvious differences at the transcript levels of the targeting snoRNAs associated with the mutated rRNA segment. U3.5 served as loading control.

**
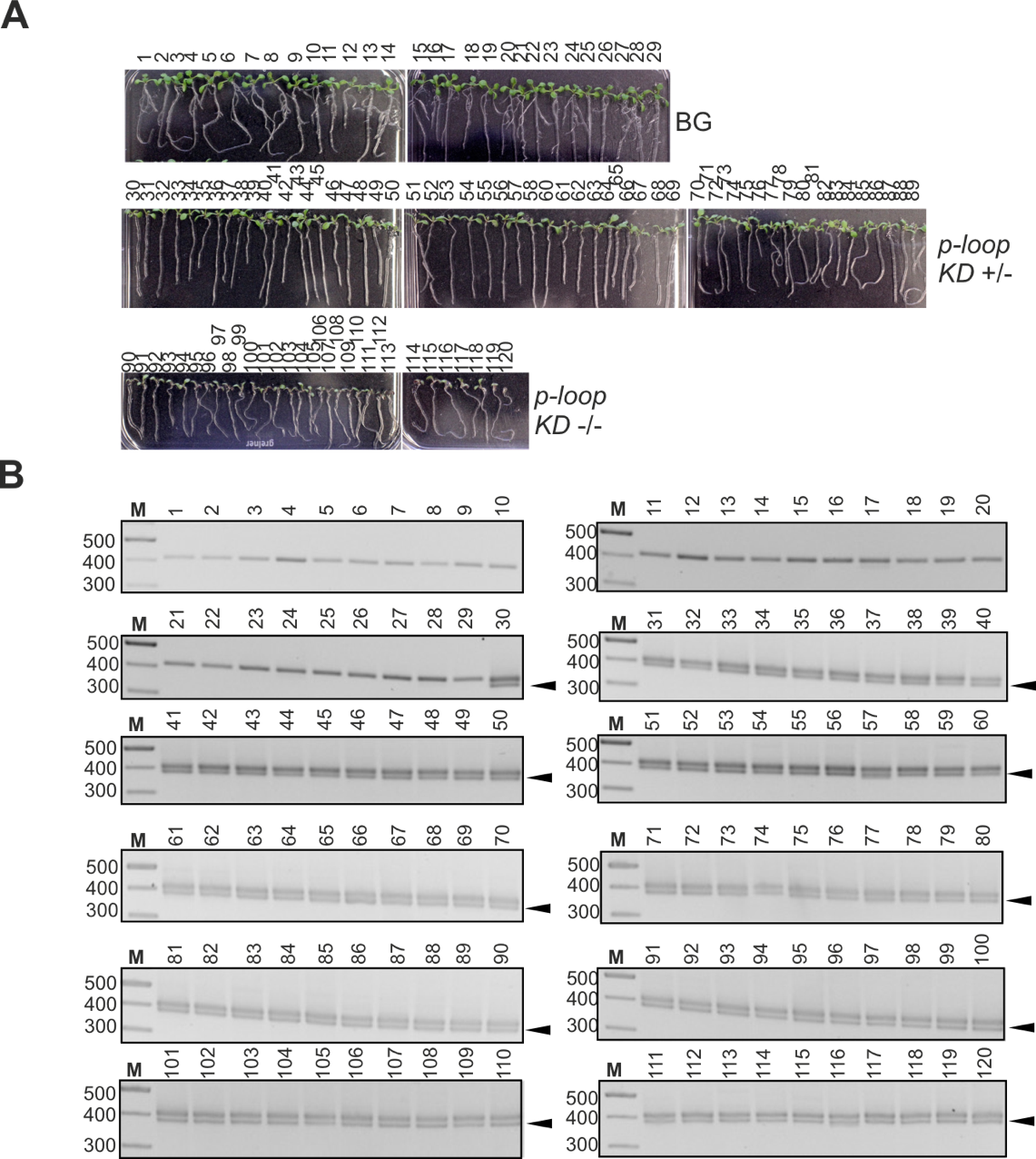
**

**Figure S3**. Genotype-phenotype correlation studies in back-crossed progenies (A) The randomized 120 progenies segregated phenotypically in 1:2:1 ratio is numbered as BG (29), *ploop KD* +/- (60) and *ploop KD* -/- (31) like seedlings. (B) PCR conducted on genomic DNA of panel A seedlings with mutation-specific forward and 25S specific reverse oligonucleotide PCR and the amplicons resolved on 2.5% TTE gel. Note the absence of mutated P-loop region lacking band (381 bp) on BG progenies. DNA marker (bp) shown on the left.


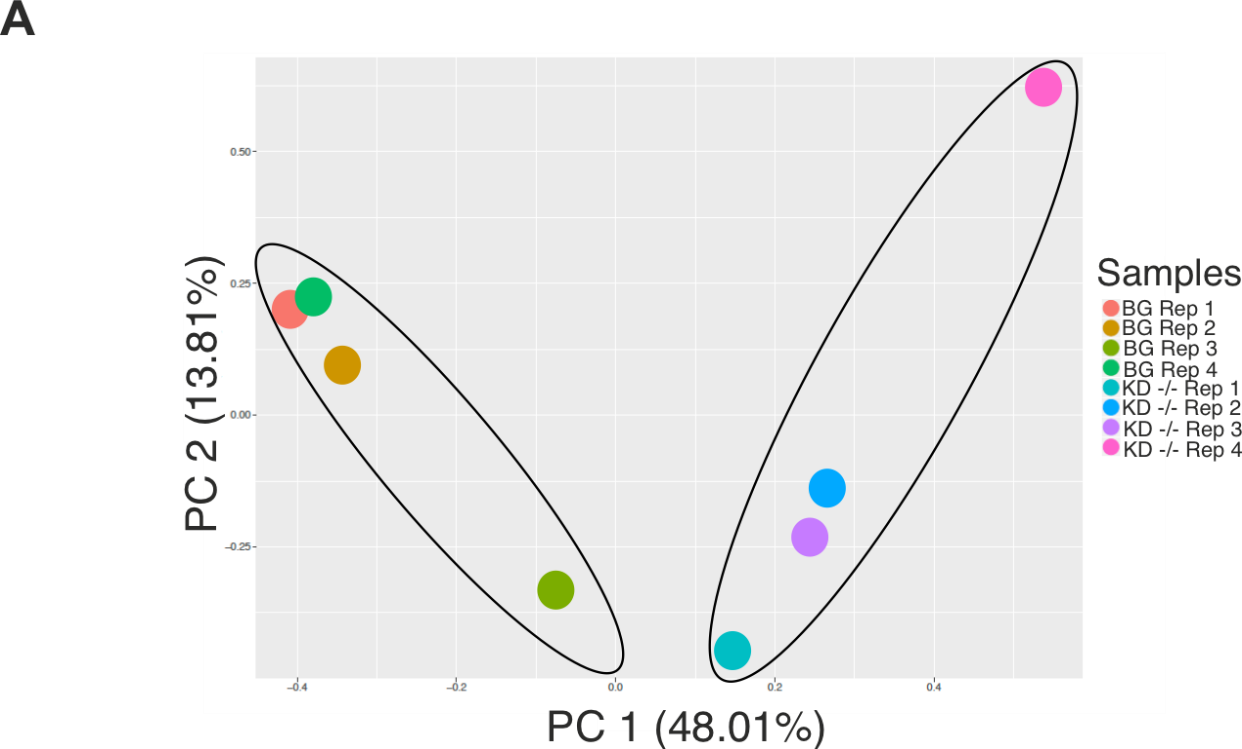


**Figure S4.** Principal component analyses of analyzed proteomes. (A) Principal component analysis of four biological replicates (denoted Rep1-4) each from BG and *ploop KD* -/- used in proteomic analysis shown in Figure 2G and H.


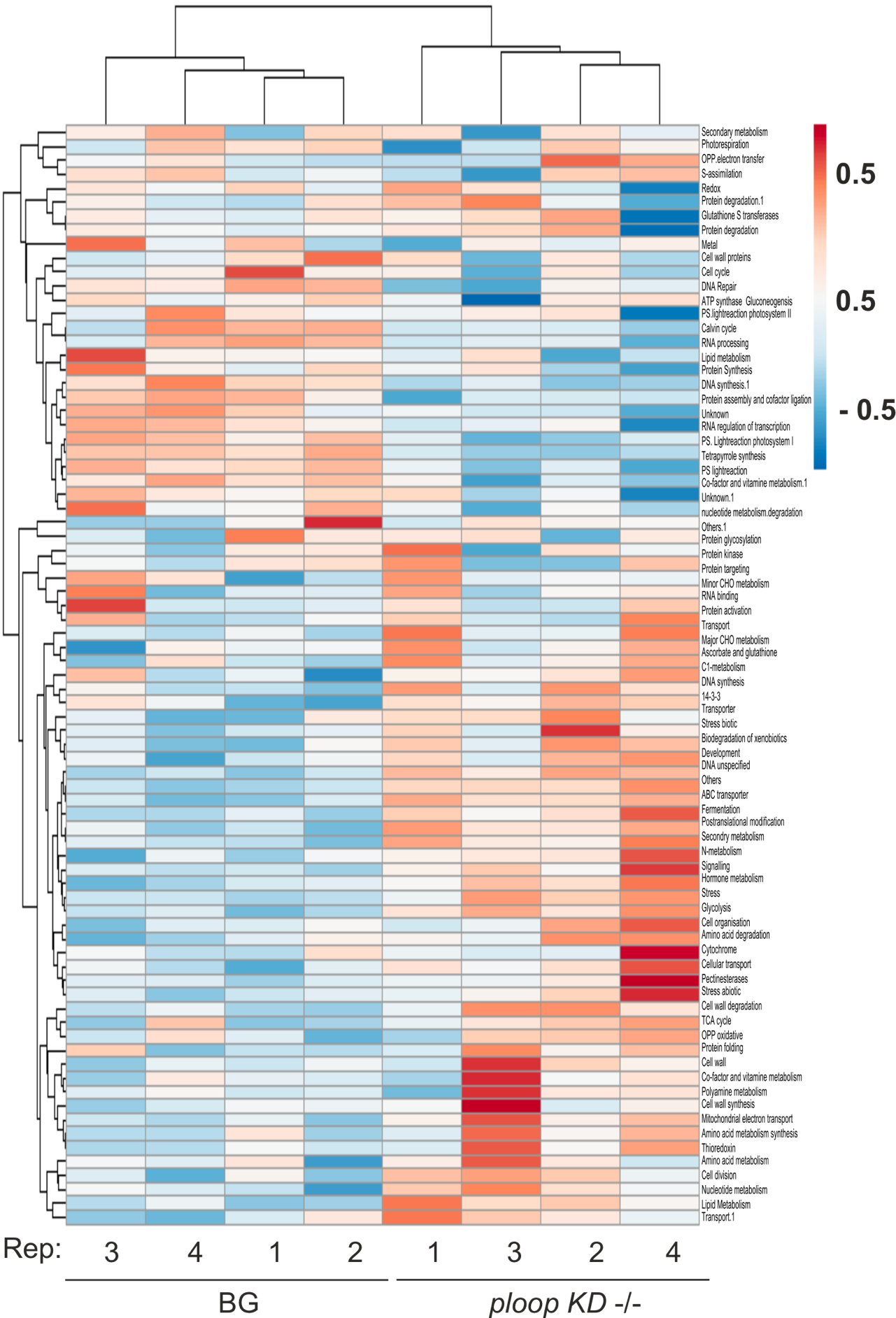


**Figure S5.** Bicluster analyses of analyzed proteomes. (A) Bicluster analysis of the abundance of the identified proteins between 4 replicates according to their GO terms. Colors in all panels allude to relative increase (red) or decrease (blue).


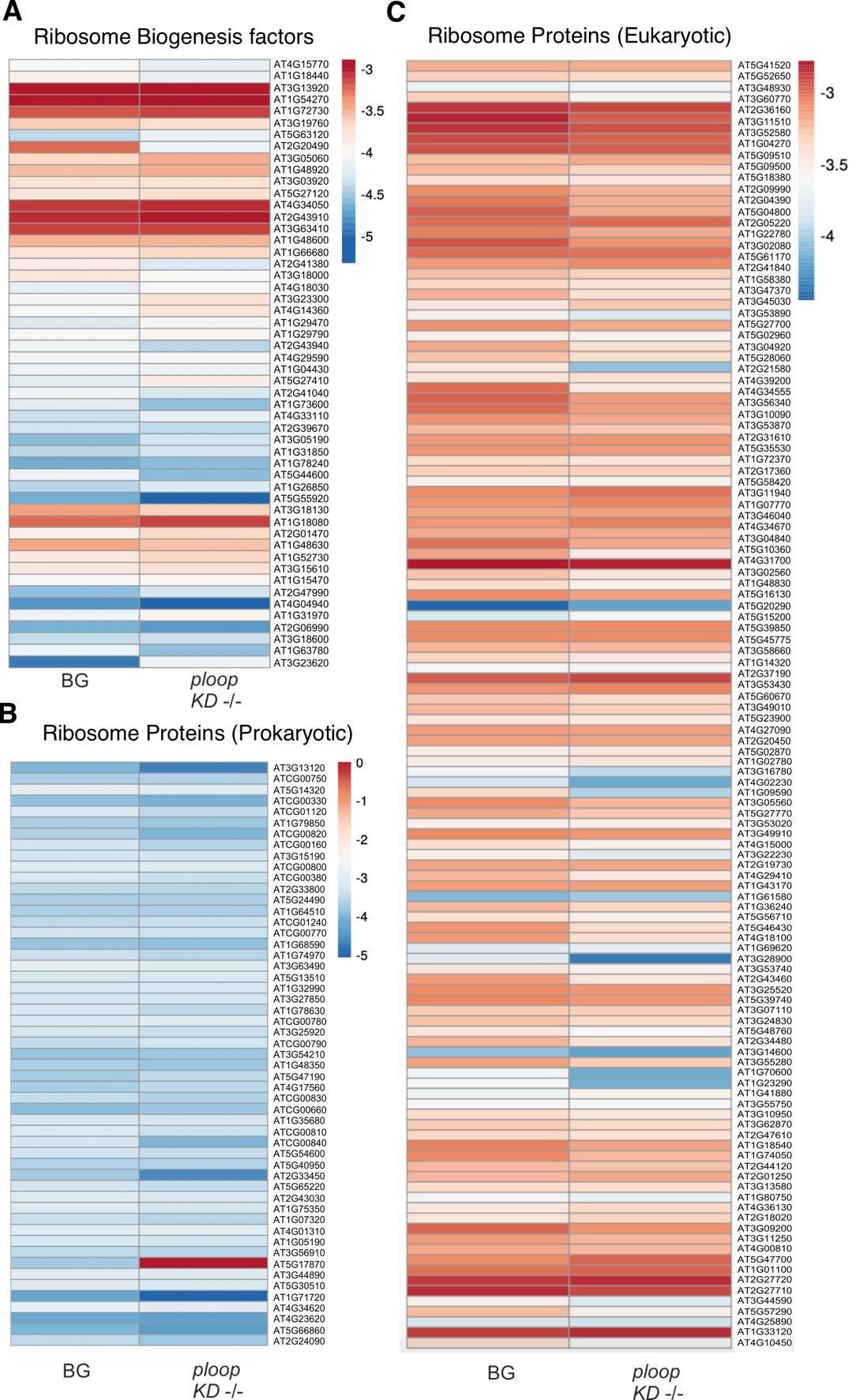


**Figure S6.** Ribosome stoichiometry changes in BG and *ploop KD* -/- mutants. The relative abundance of proteins is denoted by their respective heat maps in gene ontological category of ribosome biogenesis-related (A), prokaryotic origin ribosomal proteins (B) and eukaryotic origin ribosomal proteins (C). Colors in all panels allude to relative increase (red) or decrease (blue).

**
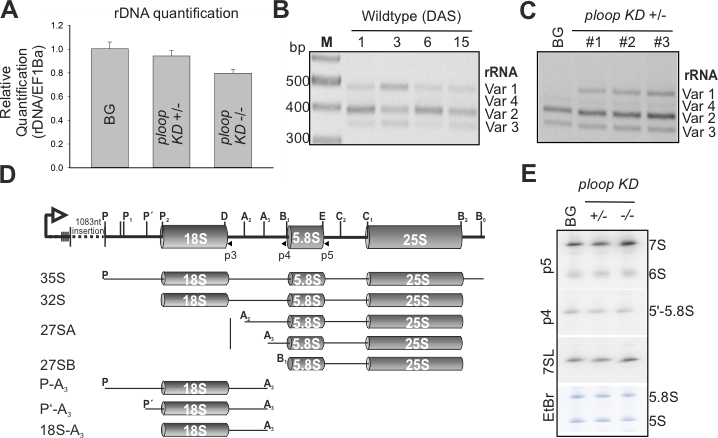
**

**Figure S7.** Complementary analyses of BG and *ploop KD* -/- plants. (A) Genomic DNA qPCR and relative quantification of rDNA copies in mutants through 5.8S amplification, normalized using EF1Ba (At5g19510) with 3 biological replicates. Error bars represent SEM. (B) rRNA VARs expression levels in WT seedlings. DNA marker (bp) shown on the left. (C) rRNA VARs expression pattern in 35 days-old apex tissues of BG and *ploop KD* +/- tissues. (D) Schematics of binding position of oligonucleotide probes (p3, p4, p5) to the pre-rRNA and the list of high molecular weight precursors identifiable after northern hybridization. (E) Northern hybridization and probing of low molecular weight pre-rRNA precursor levels in BG, *ploop KD* tissues analyzed on 10% PAGE gel using p4 and p5 probes. 7SL and EtBr staining of 5.8S and 5S served as loading control.


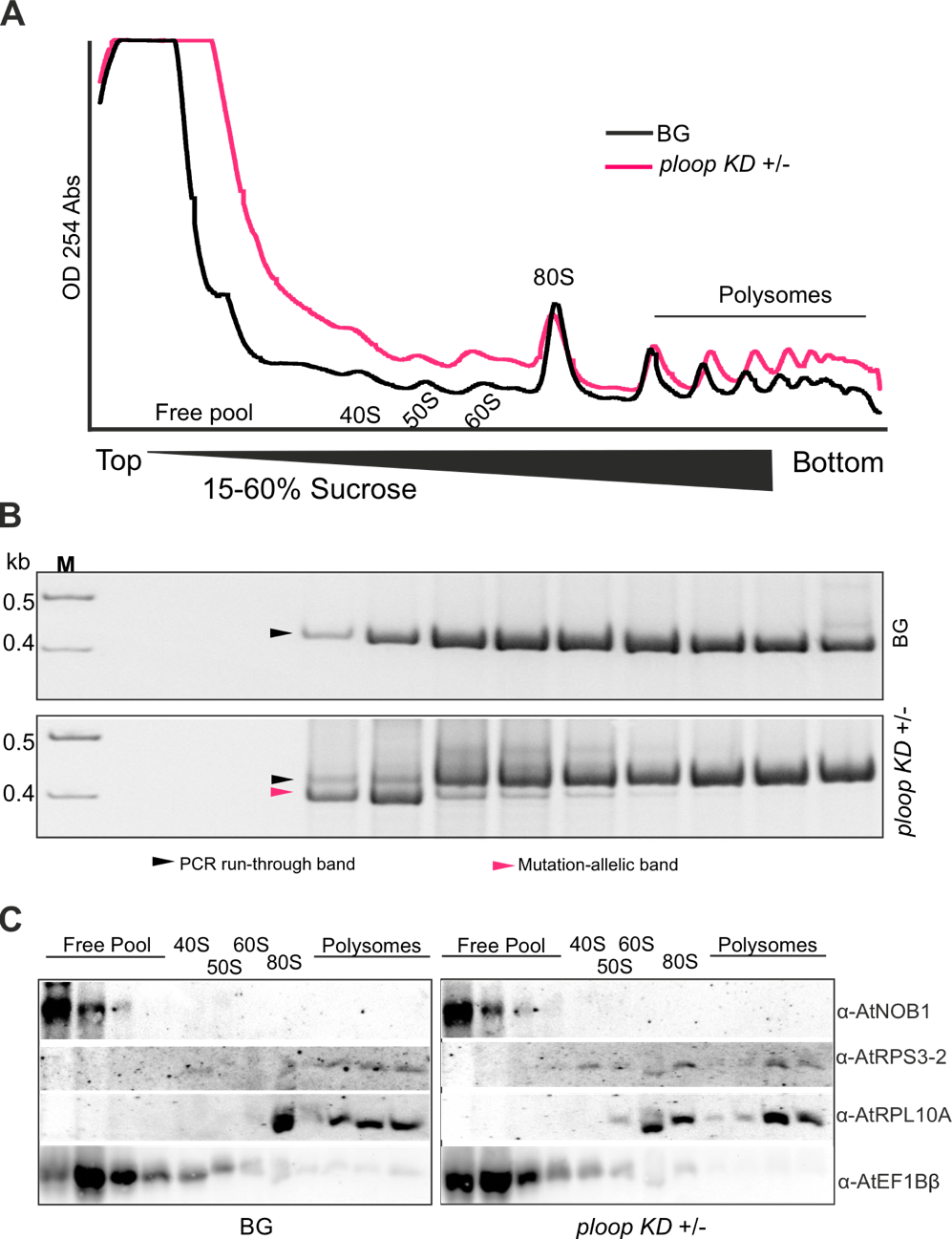


**Figure S8.** Analysis of *ploop* mutated rRNA distribution in silique tissues. (A) Absorbance profile at 254 nm of silique tissues from BG and *ploop KD* +/- plotted with time of detection from top to bottom after sucrose-density gradient centrifugation (B) The RNA from the according fractions at panel A of both genotypes purified, two nearby RNA fractions pooled, reverse transcribed and PCR conducted as described in Figure 5B. The black arrow denotes PCR-run through product and the cyan arrow indicates mutated rRNA product. (C) The protein from the fractions at panel A of both genotypes were resolved on 10% SDS-PAGE and blotted with indicated antibodies on the right.
